# Supplementary material for: Petrological and geochemical characterisation of the sarsen stones at Stonehenge
Source: PLoS One. 2021 Aug 4;16(8):e0254760. doi: 10.1371/journal.pone.0254760 (PMC8336861; doi:10.1371/journal.pone.0254760)
Supplement: Attachment — Submitted filename: Core 58 Response to reviewers R2 2021_06_04.docx [file pone.0254760.s008.docx]

**Response to Reviewer**

We thank the reviewer for his/her incredibly detailed second set of observations – even going as far as checking the supplementary data tables – that have greatly improved the manuscript. We have made changes in response to every comment, as follows:

[Reviewer] Line 25/26 “That’s a very Eurocentric statement. The Great Pyramids of Giza were built at about the same time…”

[Response] Corrected to read “…arguably the most important Late Neolithic monument in Europe”.

[Reviewer] Line 36 “omit, no need to refer to later sections in the introduction”

[Response] Corrected as suggested.

[Reviewer] Line 63/64 ““…of the Stonehenge architecture”? Reads a bit better

[Response] Corrected as suggested.

[Reviewer] Line 66 “What are hammerstones? I can guess, but be clear please”

[Response] We first use the term ‘hammerstone’ in lines 48-50. We have made minor edits to the text to expand on this information such that we do not need to repeat it later: “The hard sarsen appears to be derived from hammerstones of various size broken in the process of shaping (or dressing) the stones on site during construction.”

[Reviewer] Line 112/113 “Again, with all due respect to Stonehenge, The Great Pyramid of Khufu has already been standing for around 200 hundred years when the sarsen circles were erected…”

[Response] Corrected, as above, to read “…in Europe”

[Reviewer] Line 156-158 “Shouldn’t this be in the figure caption?” AND Line 175-176 “In the figure caption?”

[Response] We have relocated the information about informed consent into the captions for Figures 2 and 4. We trust that this is acceptable for the journal.

[Reviewer] Line 189 “36 cm”

[Response] Corrected as suggested. We have checked through the remainder of the manuscript and inserted spaces between numbers and units wherever necessary.

[Reviewer] Line 207-208 “Does that mean it had a glass cover on top?”

[Response] We have made minor edits to the text and inserted the following sentence for clarification: “Glass cover slips were not applied to either set of thin-sections”.

[Reviewer] Line 277 “No need to capitalise C and T here”

[Response] Corrected as suggested.

[Reviewer] Line 334-335 “Where? I don’t see anything mylonitic in figs 10A and B. And “mylonite” is a dynamic-metamorphic rock or process, which is probably not what happened in your rocks.”

[Response] In the interests of clarity for the reader, we have opted to delete the phrase ‘with minor mylonitic quartz’ from the text.

[Reviewer] Line 340-341 “Then why say it? Was there any reason to expect twinned feldspar?”

[Response] For brevity, we have deleted this sentence from the manuscript.

[Reviewer] Line 344-349 “References to figures?”

[Response] We have added the following sentence to the end of this paragraph: “An example of rock clast type (iii) is visible bottom centre right of Fig 10D.”

[Reviewer] Line 361 “Figures showing these things?”

[Response] We have added the following sentence to the end of this paragraph: “The characteristics of titanium mineral phases are discussed further in section 3.5.2, with examples visible in Fig 14”.

[Reviewer] Line 387 “two spaces”

[Response] Corrected as suggested and checked throughout the manuscript.

[Reviewer] Line 399 “space between number and units: 20 kV. Here and elsewhere”

[Response] Corrected as suggested and checked throughout as noted above.

[Reviewer] Line 399 “change the letter x into the multiplication symbol: ×”

[Response] Corrected as suggested and checked throughout the manuscript.

[Reviewer] Line 465 “illite-smectite appears twice here”

[Response] We have corrected this text to make it clear that there are two populations of illite-smectite present: “…illite and illite-smectite, Fe-illite and Fe-illite-smectite…”

[Reviewer] Line 505 “Having sub-nm beam precision is extremely difficult to achieve in porous material (like yours). I suggest to say “~750”. This is sufficiently precise, at 0.75 micrometre.”

[Response] Corrected as suggested.

[Reviewer] Line 515 “Your own radiation when analysing, or background geological radiation? If the former, then it would affect all quartz similarly, which obviously didn’t happen.”

[Response] Thanks for this observation. For clarification we have revised the sentence as follows: “We acknowledge, however, that CL colour shifts from blue to red with increasing radiation during analysis, and that this has almost certainly happened, to a limited extent, during the scanning of the polished sections.”

[Reviewer] Line 559 ““pXRF” is much more common term than “PXRF”

[Response] Corrected as suggested and throughout the manuscript.

[Reviewer] Line 760 “no need to capitalise HFS”

[Response] Corrected as suggested.

[Reviewer] Line 784 “no need to capitalise”

[Response] Corrected as suggested.

[Reviewer] Line 819 “1.3 ppm”

[Response] Corrected as suggested and checked throughout as noted above.

[Reviewer] Line 849 “illite-smectite is repeated”

[Response] As noted above, we have corrected this text to make it clear that there are two populations of illite-smectite present: “…illite and illite-smectite, Fe-illite and Fe-illite-smectite…”

[Reviewer] Line 859 “What is this? It’s written like it’s a term for something specific?”

[Response] We have added the following text to explain the term Clay-with-Flints for an international readership: “(a residual deposit formed from the dissolution, decalcification and cryoturbation of the Chalk Group and Palaeogene formations)”.

[Reviewer] Line 948 “tourmaline-absent”

[Response] Corrected as suggested.

[Reviewer] Line 960 “no space between number and %”

[Response] Corrected as suggested.

[Reviewer] Line 1032 “Fisher”

[Response] Corrected as suggested.

[Reviewer] Line 1230 “There’s a column called “LE” after uranium – what’s that?”

[Response] LE stands for light elements. We have added clarifying text to the bottom of the spreadsheet in S1 Data as follows: “LE indicates 'light element fraction' (atomic number <18)”.
